# Dietary xylo-oligosaccharides and arabinoxylans improved growth efficiency by reducing gut epithelial cell turnover in broiler chickens

**DOI:** 10.1186/s40104-024-00991-z

**Published:** 2024-03-04

**Authors:** Carla Castro, Shahram Niknafs, Gemma Gonzalez-Ortiz, Xinle Tan, Michael R. Bedford, Eugeni Roura

**Affiliations:** 1 Centre for Nutrition and Food Sciences, Queensland Alliance for Agriculture and Food Innovation, The University of Queensland, St Lucia, QLD 4072 Australia; 2 AB Vista, Marlborough, Wiltshire SN8 4AN UK

**Keywords:** Actin, Arabinoxylans, Broiler, Cell turnover, Energy metabolism, Jejunum, Xylo-oligosaccharides

## Abstract

**Background:**

One of the main roles of the intestinal mucosa is to protect against environmental hazards. Supplementation of xylo-oligosaccharides (XOS) is known to selectively stimulate the growth of beneficial intestinal bacteria and improve gut health and function in chickens. XOS may have an impact on the integrity of the intestinal epithelia where cell turnover is critical to maintain the compatibility between the digestive and barrier functions. The aim of the study was to evaluate the effect of XOS and an arabinoxylan-rich fraction (AXRF) supplementation on gut function and epithelial integrity in broiler chickens.

**Methods:**

A total of 128 broiler chickens (Ross 308) were assigned into one of two different dietary treatments for a period of 42 d: 1) control diet consisting of a corn/soybean meal-based diet; or 2) a control diet supplemented with 0.5% XOS and 1% AXRF. Each treatment was randomly distributed across 8 pens (*n* = 8) with 8 chickens each. Feed intake and body weight were recorded weekly. On d 42, one male chicken per pen was selected based on average weight and euthanized, jejunum samples were collected for proteomics analysis.

**Results:**

Dietary XOS/AXRF supplementation improved feed efficiency (*P* < 0.05) from d 1 to 42 compared to the control group. Proteomic analysis was used to understand the mechanism of improved efficiency uncovering 346 differentially abundant proteins (DAP) (*P*_adj_ < 0.00001) in supplemented chickens compared to the non-supplemented group. In the jejunum, the DAP translated into decreased ATP production indicating lower energy expenditure by the tissue (e.g., inhibition of glycolysis and tricarboxylic acid cycle pathways). In addition, DAP were associated with decreased epithelial cell differentiation, and migration by reducing the actin polymerization pathway. Putting the two main pathways together, XOS/AXRF supplementation may decrease around 19% the energy required for the maintenance of the gastrointestinal tract.

**Conclusions:**

Dietary XOS/AXRF supplementation improved growth efficiency by reducing epithelial cell migration and differentiation (hence, turnover), actin polymerization, and consequently energy requirement for maintenance of the jejunum of broiler chickens.

**Supplementary Information:**

The online version contains supplementary material available at 10.1186/s40104-024-00991-z.

## Introduction

As the demand for poultry meat continues to grow worldwide, the industry sets goals that drives towards improving not only productivity, but also the health and welfare of chickens. Optimizing gut health has become a primary focus especially since the restrictions on the use of antibiotics in farm animals in European countries were introduced and later adopted by many countries worldwide [1, 2]. Intestinal health is supported by an intricate network of interactions between the microbiota, physiological functions, immune response, and morphological integrity, all of them critical for the general health and performance of chickens.

Significant resources have been invested to study alternatives to in-feed antibiotics, including the use of exogenous enzymes, phytogenic compounds, organic acids, probiotics and prebiotics amongst others [3–10]. Xylo-oligosaccharides (XOS) have been reported to exert a prebiotic effect helping develop a healthy microbiota and have become one of the most commonly used oligosaccharides in the poultry industry. They are composed of xylose units linked by β-1,4-glycosidic bonds [11]. XOS can be generated by the action of endo-β-1,4-xylanases on arabinoxylan (AX), resulting in the production of arabinoxylo-oligosaccharides and non-substituted XOS [11, 12]. However, chicken endogenous enzymes cannot degrade AX which allows them to reach the hindgut intact where microbial fermentation occurs. XOS have shown to positively impact the gut microbiota, enhance short-chain fatty acid (SCFA) production, stimulate immune activity in the gastrointestinal tract, and improve energy utilisation of cereals resulting in improved growth efficiency in chickens [13–18].

There is evidence showing a beneficial role of dietary XOS on gut function associated with the enhancement of the intestinal epithelial barrier integrity [19–21]. The intestinal barrier protects the host from pathogens and antigens in the intestinal lumen. It is formed by a monolayer of polarised cell types that migrate, proliferate and differentiate, to later be shed into the lumen as part of a physiological process that requires a high cell turnover to sustain homeostasis and epithelial integrity [22, 23]. This process requires significant energy expenditure to maintain proper functioning [24, 25]. In addition, gut epithelial stressors such as inadequate nutrition, a disease challenge, or an inflammatory process, increases the demand for a rapid cell turnover to maintain the barrier function. Failure to keep up with the increased demand of renewal of epithelial cells may result in loss of epithelial integrity and increased intestinal permeability also referred to as leaky gut [26].

Currently there is no evidence of the effect of dietary XOS on the biological mechanisms involved in intestinal integrity. Therefore, this research aimed at uncovering the effects of XOS and AX on gut function and the subsequent molecular changes involved in epithelial integrity in the small intestine of broiler chickens. It was hypothesised that XOS and AX supplementation would improve gut health and reduce energy requirements associated with epithelial cell renewal in the intestine.

## Materials and methods

### Experimental design and dietary treatments

This experiment, protocol and practices were approved by the Production and Companion Animals - Animal Ethics Committee (AEC) of The University of Queensland, Australia (approval number 2020/AE000411).

A total of 128 one-day-old Ross 308 broiler chickens of mixed sexes were purchased from Darwalla Group (QLD, Australia). Chickens were housed at the Queensland Animal Science Precinct (QASP) of The University of Queensland (Gatton Campus QLD, Australia). On arrival birds were weighed and randomly allocated into 16 floor pens of 1.1 m^2^ (8 chickens/pen). The pens had been prepared with 5-cm of wood shaving covered with 2 layers of papers. Each pen was equipped with one nipple drinker and a hopper feeder. Water and feed were provided ad libitum for the entire experimental period (42 d).

Two dietary treatments were tested: 1) a control or standard diet consisting of corn/soybean meal-based mashed diet; and 2) a control diet supplemented with 0.5% XOS (95% purity) plus 1% arabinoxylan-rich fraction (AXRF) from wheat (containing approximately 30% AX; Penfords Corporation, USA) [27]. Each treatment was randomly allocated across 8 pens (*n* = 8) for a total of 128 chickens. All diets were formulated to meet or exceed the requirements in three phases: starter from d 0 to 14; grower from d 14 to 28; and finisher from d 28 to 42 (Table 1).

**Table 1:** Ingredient and nutrient composition of control and supplemented broiler diets^a^

**Item**	**Starter (d 0 to 14)**	**Grower (d 14 to 28)**	**Finisher (d 28 to 42)**
Ingredients, % (as-fed basis)			
Maize	63.68	68.54	70.35
Soy hull	-	-	2.5
Soyabean meal	24.85	20.80	17.30
Meat and bone meal	8.6	7.6	6.9
Recycled veg oil-mixed	1.0	1.0	1.0
Limestone bag-tip	0.75	1.0	1.0
Sodium bicarbonate m	0.05	0.05	0.00
Salt-micro	0.20	0.20	0.25
Lysine HCl	0.247	0.221	0.140
DL-Methionine	-	0.204	0.180
Threonine	-	-	0.055
Vitamin premix	0.395	0.375	0.307
Calculated composition, % (as-fed basis)			
Dry matter	88.62	88.50	88.45
Crude protein	22.03	20.06	18.50
Crude fibre	2.54	2.45	3.20
Crude fat	4.70	4.72	4.71
Metabolizable energy, MJ/kg	12.85	13.00	12.81
L-Lysine	1.30	1.15	1.00
Methionine	0.61	0.56	0.50
Tryptophan	0.23	0.21	0.19
Calcium	1.47	1.43	1.35
Phosphorus	0.90	0.83	0.78

^a^Control diet consisted of a standard broiler feed. Supplemented feed consisted of the control diet described in the table supplemented with 0.5% of xylo-oligosaccharides and 1% arabinoxylan-rich fraction

### Performance parameters and sample collection

Body weight and feed intake were measured per pen weekly from d 0 to 42. Average daily gain (ADG), average daily feed intake (ADFI) and feed conversion ratio (FCR) were calculated for each of the 3 phases and the overall. Mortality was recorded together with feed disappearance and the calculations of the performance parameters compensated accordingly. At d 42, one male chicken per pen was randomly selected based on average weight and euthanised using cervical dislocation. Birds were confirmed to be male by post-mortem examination. Jejunum samples (1 g) were collected and placed in 1.5 mL of RNA-later solution and stored at −80 °C until analysis was performed.

### Protein sample preparation, digestion, and high pH fractionation for proteomics analysis

Initially samples were denatured and alkylated [28, 29]. Briefly, 20 mg of jejunum sample were added into 1 mL of guanidine denaturing buffer (6 mol/L Guanidine Chloride (GndCl), 10 mmol/L Dithiothreitol (DTT) and 50 mmol/L Tris-HCl), homogenised and later incubated for 1 h at 30 °C using a thermomixer (Eppendorf Thermomixer^®^ C). This procedure was followed by the addition of acrylamide to a final concentration of 30 mmol/L to alkylate cysteines and incubated at 30 °C for 1 h. To quench the excess of acrylamide, DTT was added (10 mmol/L). Then proteins were quantified using acetone protein precipitation. This process was performed by adding four times the volume of acetone to one volume of sample and incubated for 60 min at −20 °C, and later centrifuged at 15,000 × *g* for 10 min. Supernatant was disposed and the dried pellet resuspended in 100 mmol/L Tris-HCl, proteins were quantified using NanoDrop^TM^ (Thermo Fisher Scientific, Waltham, USA).

Lysate containing 100 µg total protein were processed with Filter Assisted Sample Preparation (FASP) for trypsin digestion using 10 kDa Cut-off Amicon columns and centrifugated at 14,000 × *g* for 40 min [30]. A volume of 500 μL of 100 mmol/L ammonium acetate was added to the filter and centrifuged at 14,000 × *g* for 20 min. This step was repeated twice to wash the samples. Following centrifugation, samples were diluted in 100 µL of 100 mmol/L ammonium acetate. Trypsin (proteomics grade, Sigma-Alldrich, USA) was added to the top of the column at a ratio of 1:50 (enzyme:protein) and incubated overnight at 37 °C, followed by centrifugation at 14,000 × *g* for 40 min. A total of 50 µL of NaCl (50 mmol/L) was used to wash the columns and centrifuged at 14,000 × *g* for 10 min. The filtrate obtained from centrifugation contained the digested peptides. Filtrate containing the digested peptides were then desalted with C-18 Zip-tips (Merck Millipore, Cork, Ireland) and eluted in 90% acetonitrile (ACN) and 0.1% formic acid (FA). Then samples were dried in a vacuum concentrator (MiVac Quattro concentrator, Genevac Ltd., Ipswich, UK) at 45 °C for 30 min. Dried peptides were resuspended in 100 µL of 1% ACN/0.1% FA. In addition, a pooled sample containing approximately 40 μg of peptides in a final volume of 500 μL in 0.1% FA were subjected to high pH reverse-phase fractionation [28]. Peptides were added to Sep-Pak vac tC18 cartridges (Waters, Milford, MA, USA) and washed with 500 μL of Milli-Q water, and then eluted in eight separated fractions containing 500 μL of solution containing ACN at 5%, 7.5%, 10%, 12.5%, 15%, 17.5%, 20%, or 50% in 0.1% of triethylamine and then placed in a vacuum concentrator at 45 °C overnight. The next morning, samples were resuspended in 0.1% FA and sent for mass spectrometry (MS) analysis as described below.

### Mass spectrometry and data analysis

Samples were separated using reversed-phase chromatography on a Prominence nanoLC system (Shimadzu, Kyoto, Japan) [28, 31], with some modifications as described below. Using a flow rate of 30 µL/min, samples were desalted on an Agilent C18 trap (0.3 mm × 5 mm, 5 µm) for 3 min, followed by separation on a Vydac Everest C18 (300 A, 5 µm, 150 mm × 150 µm) column at a flow rate of 1 µL/min. LC gradient was: 5%–35% B over 45 min, 35%–60% B over 7 min, 60%–97% B over 1 min, held at 97% B for 5 min, 97%–5% B over 2 min, and column re-equilibrated for 7 min. Buffer A = 1 % ACN/0.1% FA and buffer B = 80% ACN/0.1% FA. Eluted peptides were directly analysed on a TripleTOF 5600 instrument (AB Sciex) using a Nanospray III interface. Gas and voltage settings were adjusted as required. For data dependent acquisition (DDA) analyses, a MS-TOF scan across 350–1,800 *m/z* was performed for 0.5 s followed by information dependent acquisition of up to 20 peptides with intensity greater than 100 counts, across 40–1,800 *m/z* (0.05 s/spectra) using collision energy (CE) of 40 ± 15 V. For Sequential Window Acquisition of all Theoretical Mass Spectra (SWATH-MS) analyses, all LC-MS parameters were the same, except MS scans across 350–1,800 *m/z* were performed (0.05 s), followed by high-sensitivity data independent acquisition (DIA) mode using 26 *m/z* isolation windows for 0.1 s, across 400–1,250 *m/z*. CE values for SWATH samples were automatically assigned by Analyst software (SCIEX) based on *m/z* mass windows.

Proteins were identified using information-dependent acquisition analysis (DDA data) with Protein Pilot v5.0.1 (SCIEX, Concord, Canada). The database used by Protein Pilot was downloaded from Uniprot (www.uniprot.org – downloaded August 2021). False discovery rate was conducted with limits of 99% confidence and 1% local false discovery rate. SWATH-MS relative quantitative proteomics data was analysed with PeakView v2.1 (AB Sciex) software.

### Functional enrichment analysis

The differentially abundant proteins (DAP) between control and XOS supplemented chickens were used as an input for functional enrichment analysis. Database for Annotation Visualization and Integrated Discovery (DAVID) 6.8 Bioinformatic resources was used to identify biological pathways enriched by DAP [32, 33]. Gene Ontology terms (GO), Kyoto Encyclopedia of Genes and Genomes (KEGG) and REACTOME databases, were used to attain insight into the functions of proteins. The target list of DAP was compared to the background list of the 1,161 proteins that were identified. Significance was determined at a *P*-value < 0.05.

### Statistical analysis

Performance data of XOS/AXRF supplemented and control chickens were analysed by *t*-Student test using R software (RStudio, Inc., Boston, Massachusetts, USA). Data are expressed as mean ± standard error (SE). A *P*-value < 0.05 was considered statistically significant. Pen averages were used as the experimental unit.

Statistical analysis of proteomics data was performed using MSstats (2.4) in R software to identify DAP with a *P*-value lower than 0.00001 adjusted [34].

## Results

### Performance parameters

Dietary XOS/AXRF supplementation significantly (*P* < 0.05) improved FCR when compared with the control group during the grower (1.62 vs. 1.73), finisher (1.87 vs. 2.02), and the overall (1.73 vs. 1.82) periods (Table 2). No significant effects were observed on ADFI and ADG.

**Table 2:** Effect of combining xylo-oligosaccharides and arabinoxylan-rich fraction (XOS/AXRF) on growth performance at different dietary phases

**Period**	**Parameter**	**Control**	**± SE**	**XOS/AXRF**	**± SE**	***P*-value**
Starter (d 1–14)	ADG, g	24.6	0.54	24.1	0.62	0.5775
	ADFI, g	34.4	1.79	36.4	1.06	0.3568
	FCR, g/g	1.40	0.06	1.51	0.05	0.1539
Grower (d 14–28)	ADG, g	63.6	0.63	64	1.14	0.8020
	ADFI, g	110	2.34	104	1.78	0.0601
	FCR, g/g	1.73^a^	0.04	1.62^b^	0.02	0.0454
Finisher (d 28–42)	ADG, g	83.9	1.62	86.3	1.58	0.3051
	ADFI, g	169.2	4.30	161.4	3.11	0.1625
	FCR, g/g	2.02^a^	0.04	1.87^b^	0.01	0.0012
Overall (d 1–42)	ADG, g	57.4	0.69	58.1	0.87	0.5056
	ADFI, g	104.5	2.18	100.6	1.60	0.1666
	FCR, g/g	1.82^a^	0.04	1.73^b^	0.01	0.0264
	Mortality, %	1.56	1.56	1.56	1.56	1.0000

*Control* Corn/soybean meal-based diet, *XOS/AXRF* Control diet supplemented with 0.5% of XOS and 1% AXRF. *ADG* Average daily gain, *ADFI* Average daily feed intake, *FCR* Feed conversion ratio, *SE* Standard error

^a,b^Different superscripts in the same row differ significantly at* P* < 0.05 level in *t*-Student test

### Protein identification and pathway analysis

**Fig. 1:**
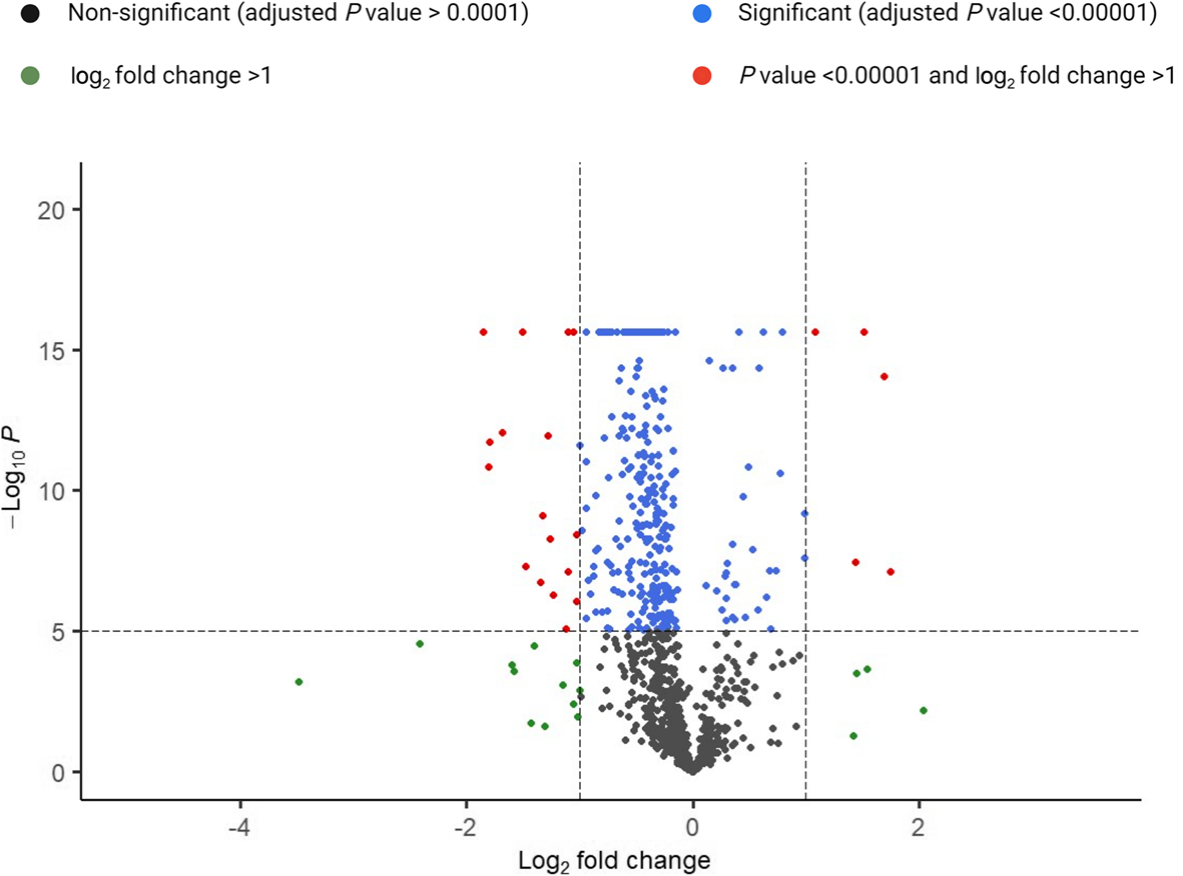
Volcano plot of differentially abundant proteins in chickens fed xylo-oligosaccharides and arabinoxylan-rich fraction versus control. The *Y*-axis is the negative log of the *P* value with base 10, and *X*-axis is the log of the fold-change with base 2. Xylo-oligosaccharide and arabinoxylan-rich fraction supplemented at 0.5% and 1% level, respectively

A total of 1,161 proteins were identified, of which 346 (30%) were differentially abundant (*P* < 0.00001) when comparing the XOS/AXRF supplemented to the control chickens. From the total DAP, 36 showed significantly higher abundance and 310 showed lower abundance in the jejunum of chickens that received XOS/AXRF versus control (Fig. 1). LOC100859645, CYP3A4, TPM1, PKP2, and SAR1B had the lowest abundance in XOS/AXRF fed chickens. Whereas CKB, STXBP5L, COPA, SEC23, and RPS7 presented the highest abundance in the same group of chickens.

**Fig. 2:**
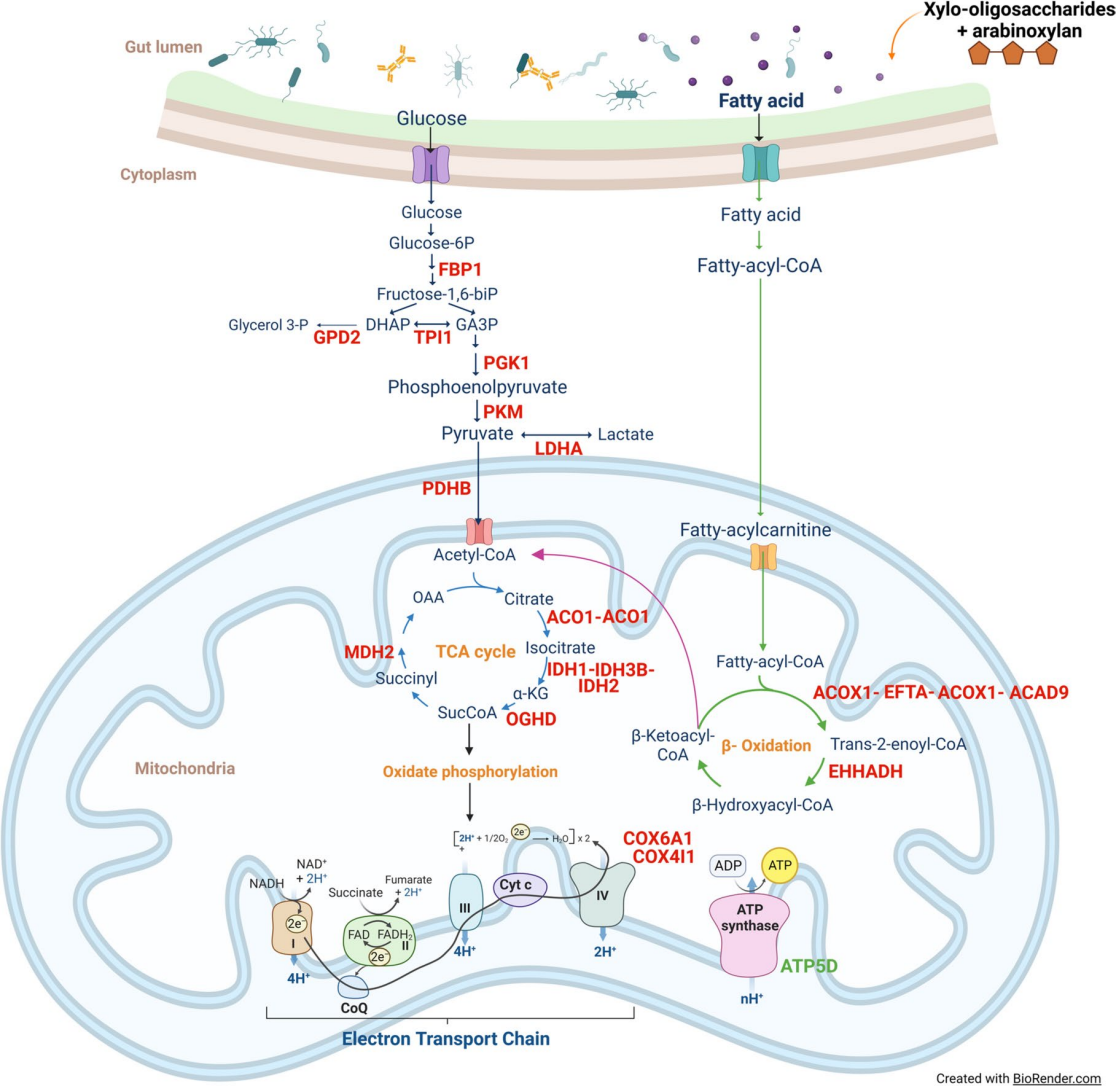
Differentially abundant proteins involved in cell metabolism in the jejunum of supplemented versus control chickens. Xylo-oligosaccharide and arabinoxylan-rich fraction supplemented at 0.5% and 1% level, respectively. In red, proteins showing lower abundance in XOS/AXRF supplemented chickens. In green, proteins showing higher abundance in XOS/AXRF supplemented chickens. *FBP1* Fructose-1,6-bisphosphatase 1, *GDP2* Glycerol-3-phosphate dehydrogenase 2, *TPI1* Triosephosphate isomerase 1, *PGK1* Phosphoglycerate kinase 1, *PKM* Pyruvate kinase, *LDHA* Lactate dehydrogenase A, *PDHB* Pyruvate dehydrogenase (lipoamide) beta, *ACO1* Aconitase 1, soluble, *ACO2* aconitase 2, *IDH1* Isocitrate dehydrogenase 1 (NADP^+^), soluble, *IDH2* Isocitrate dehydrogenase 2 (NADP^+^), mitochondrial, *IDH3B* Isocitrate dehydrogenase 3 (NAD^+^) beta, *MDH2* Malate dehydrogenase 2, *ACOX1* Acyl-CoA oxidase 1, palmitoyl, *ACAD9* Acyl-CoA dehydrogenase family, member 9, *EHHADH* Enoyl-CoA hydratase and 3-hydroxyacyl CoA dehydrogenase, *COX6A1* Cytochrome c oxidase subunit VIa polypeptide 1, *COX4I1* Cytochrome c oxidase subunit IV isoform 1

The differential enrichment analysis uncovered several metabolic pathways enriched (*P* < 0.05) by DAP in the jejunum of chickens that received XOS/AXRF compared to control when GO, KEGG and REACTOME databases were used (Additional file 1: Table S1). This list of proteins translated into a decreased activity of numerous pathways involved in energy metabolism (Fig. 2) and actin dynamics (Table 3) associated with cell migration.

**Table 3:** Differentially abundant proteins (DAP) and actin-related biological pathways in chickens fed xylo-oligosaccharide and arabinoxylan-rich fraction^a^

Database	Pathway	*P*-value	Downregulated (↓)/ upregulated (↑) DAP
Gene Ontology	Actin filament polymerization	0.0000	↓ RAC1, ARPC4, COTL1, VIL1, GSN, CTTN
	Barbed-end actin filament capping	0.0015	**↓** HSPB1, VIL1, GSN, CAPZA2
	Actin filament severing	0.0046	**↓** VIL1, GSN; ↑ DSTN
	Actin cytoskeleton reorganization	0.0092	**↓** RAC1, MYH9, CTTN, EZR, ANXA1
	Actin filament-based movement	0.0191	**↓** MYH9, MYO6, MYO1A
	Regulation of cell-cell adhesion mediated by integrin	0.0433	**↓** ADA; ↑ DPP4
	Epithelial cell differentiation	0.0499	**↓** ANXA4, BDH2, VIL1, HNRNPH3
Reactome	Gap junction degradation	0.0328	**↓** MYO6, AP2M1, CLTC
	RHO GTPases activate PAKs	0.0402	**↓** RAC1, CDC42, MYLK
	RHO GTPases Activate WASPs and WAVEs	0.0464	**↓** RAC1, CDC42, ARPC4, ACTR2

^a^Xylo-oligosaccharide and arabinoxylan-rich fraction supplemented at 0.5% and 1% level, respectively. *RAC1* Ras-related C3 botulinum toxin substrate 1, *ARPC4* Actin-related protein 2/3 complex, subunit 4, *COTL1* Coactosin-like F-actin binding protein 1, *VIL1* Villin 1, *GSN* Gelsolin, *CTTN* Cortactin, *HSPB1* Heat shock protein family B (small) member 1, *CAPZA2* Capping actin protein of muscle Z-line alpha subunit 2, *DSTN* Destrin, actin depolymerizing factor, *MYH9* Myosin, heavy chain 9, non-muscle, *EZR* Ezrin, *ANXA1* Annexin 1, *MYO6* Myosin 6, *MYO1A* Myosin IA, *ADA* Adenosine deaminase, *DPP4* Dipeptidyl peptidase 4, *ANXA4* Annexin A4, *BDH2* 3-Hydroxybutyrate dehydrogenase 2, *HNRNPH3* Heterogeneous nuclear ribonucleoprotein H3, *AP2M1* Adaptor-related protein complex 2 mu 1 subunit, *CLTC* Clathrin heavy chain, *CDC42* Cell division cycle 42, *MYLK* Myosin light chain kinase, *ACTR2* ARP2 Actin-related protein 2 homolog

On energy metabolism (Fig. 2), the KEGG database showed several pathways associated to energy metabolism that were significantly downregulated including ‘oxidative phosphorylation’, ‘glycolysis/gluconeogenesis’, and ‘fatty acid degradation’. In GO, the downregulated pathways were ‘tricarboxylic acid cycle’, ‘oxidation-reduction process’, ‘gluconeogenesis’, ‘fatty acid beta-oxidation using acyl-CoA dehydrogenase’, ‘ATP synthesis coupled proton transport’, and ‘fatty acid beta-Oxidation’. Finally, using the REATOME database the downregulation of the ‘gluconeogenesis’ and ‘glycolysis’ pathways was identified (Fig. 2).

Regarding actin dynamics (Table 3), the main pathways identified by GO included “actin filament polymerization” (*P* < 0.0001), “barbed-end actin filament capping” (*P* < 0.002), and “acting filament severing” (*P* < 0.01) to highlight only the top three. Other pathways identified (*P* < 0.05) relevant to mention are “regulation of cell-cell adhesion mediated by integrin”, and “epithelial cell differentiation”. Using the REACTOME database the “gap junction degradation” pathway can be highlighted with the highest significance (*P* < 0.05).

## Discussion

The main point in the results reported in this manuscript is that inclusion of XOS plus AXRF improved growth efficiency of broiler chickens associated with a decrease in gut epithelial cell maturation and migration. The maintenance of a healthy gut epithelia accounts for roughly 20% of the energy and protein synthesis requirements [25, 35–39]. The supplementation of XOS/AXRF improved feed efficiency by 5% suggesting that the reduced intestinal cell turnover decreased the energy requirement for maintenance of the gastrointestinal tract (GIT) by 11.7 kcal/chick/d calculated based on the metabolizable energy of the diet and the difference in feed intake. This, in turn, represents a decrease in 19% in the energy of maintenance used by the GIT in the XOS/AXRF group compared to the control. Several studies have reported the impact of dietary XOS supplementation on improving growth performance in chickens [14, 17, 40–44]. This positive effect has been associated with their capacity to modulate the gut microbiota, regulate the immune function, and enhance gut health [16, 19, 45, 46]. In addition, AX supplementation have also been reported in the literature [47–49]. Morgan et al. [15] reported that arabinoxylo-oligosaccharides supplementation improved energy utilization in broilers suggesting that this improvement was related to a prebiotic effect. This is compatible with the findings in this work pointing at a healthier gut as indicated by the impact of XOS lowering epithelial cell migration and energy expenditure in the jejunum.

Proteome analysis of the jejunum in XOS/AXRF supplemented chickens showed that DAP were related to reduced activity in biological pathways involved in cell metabolism, epithelial cell differentiation, and actin activity relevant to cell migration along the intestinal villus. These results are consistent with previous reports where dietary XOS shown a favourable impact on intestinal morphology and enhancement of the intestinal epithelial barrier function [50, 51]. The intestine has a high rate of cell renewal (including differentiation, maturation and migration of cells), which requires a large amount of energy (in the form of ATP) mainly obtained from glycolysis and mitochondrial oxidative phosphorylation [52, 53]. In the cell, the TCA cycle takes place in the mitochondria where pyruvate is oxidised leading to the production of electron donors and reducing factors utilised by the electron transport chain, hence driving ATP synthesis. An increase in mitochondrial ATP production has been associated with the increased energy demand of incremental cell migration and the promotion of wound repair [54, 55]. In contrast, our results indicate that XOS/AXRF supplementation of broiler diets reduced the ATP demand in jejunal epithelial cells, with lower abundance of critical proteins (e.g., pyruvate kinase) participating in the central metabolic pathways. Hence, it appears that XOS/AXRF fed birds have a lower need for ATP to maintain epithelial integrity compared to controls. Regarding cell differentiation, there was a reduced abundance of proteins like annexin A4 (ANXA4), a calcium/phospholipid-binding protein that has been described to be upregulated upon cell differentiation and pathologic events in the intestine [56].

**Fig. 3:**
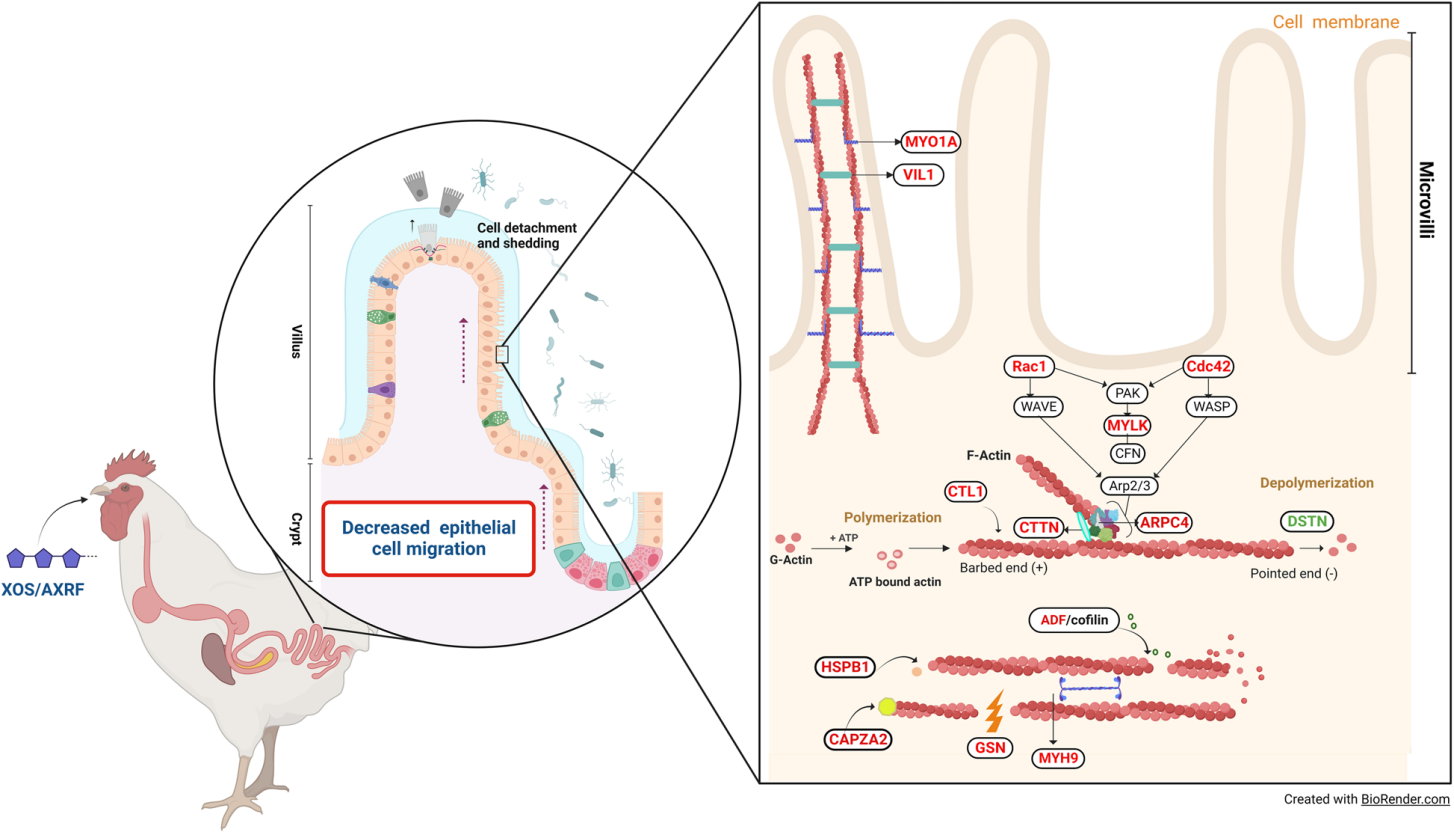
Xylo-oligosaccharide supplementation and arabinoxylan-rich fraction (XOS/AXRF) supplementation reduced intestinal cell migration associated with actin dynamic. Xylo-oligosaccharide and arabinoxylan-rich fraction supplemented at 0.5% and 1% level, respectively. In red, differentially abundant protein (DAP) showing lower abundance in XOS/AXRF supplemented chickens. In green, DAP showing higher abundance in XOS/AXRF supplemented chickens. *MYO1A* Myosin IA, *VIL1* Villin 1, *RAC1* Ras-related C3 botulinum toxin substrate 1, *ARPC4* Actin-related protein 2/3 complex, subunit 4, *COTL1* Coactosin-like F-actin binding protein 1, *GSN* Gelsolin, *CTTN* Cortactin, *HSPB1* Heat shock protein family B (small) member 1, *CAPZA2* Capping actin protein of muscle Z-line alpha subunit 2, *DSTN* Destrin, actin depolymerizing factor, *MYH9* Myosin, heavy chain 9, non-muscle, *CDC42* Cell division cycle 42, *MYLK* Myosin light chain kinase, *PAK* P21-activated kinase, *WASP* Wiskott-Aldrich syndrome protein, *WAVE* WASP family Verprolin-homologous protein, *ARP2/3* Actin-related protein 2/3 complex

Cell migration is an active process critical for adequate cell turnover in the intestine, where cells move together and actin protrusions are directed towards the tip of the villus [57]. This process involves forces created by cell adhesions controlled by the assembly of actin to the cell membrane and regulated by the Rho family of small GTPases [58]. It is known that CDC42 and Rac1 proteins from the Rho proteins influence cell motility/migration due to its interaction with the cytoskeleton and the formation of protrusive structures [59, 60]. Healing requires migration of cells, which in turn requires the organization and enhanced activity of a number of cellular components including the cytoskeleton, a process known as intestinal restitution [61–63]. Cytoskeletal reorganisation is necessary for intestinal epithelial cell mobilisation involving the hydrolysis of ATP, which has been reported to account for almost 20% of the energy expenditure of the intestine [64, 65]. Based on this, XOS/AXRF fed chickens would utilise around 12.3 kcal/d in cytoskeleton dynamics compared to 12.8 kcal/d in non-supplemented chickens. Reorganization of the cytoskeleton involves the polymerisation and depolymerisation of actin in a reversible process known as treadmilling, where globular actin (G-actin) is added to the barbed-end of a filamentous actin (F-actin) and disassembled from the pointed-end of the filament [66]. In this study, several proteins involved in the formation and activity of microfilamentous structures, including gelsolin, an actin depolymerizing protein, and ARPC4, a subunit of the Arp2/3 complex that has an important role in polymerization of F-actin and reorganization of the cytoskeleton, showed lower abundance in XOS/AXRF fed birds [67–70]. The protein actin is the major constituent of the cytoskeleton, a crucial component regulating movement of epithelial cells [71]. The results of the study indicate that actin cytoskeletal reorganisation was reduced in XOS/AXRF chickens, while proteins like villin and non-muscular myosin II, both participating in epithelial cell migration upon injury, were downregulated [63, 72–75]. In addition, this study showed decrease dynamics of actin in jejunal cells, including changes in polymerization, depolymerization, and turnover of the protein. This, in turn, indicates a reduction of cell migration along the villous-crypt axis in chickens that received XOS/AXRF in the diet (Fig. 3). To the best of our knowledge, this is the first time that dietary XOS and AXRF combined supplementation has been associated with mechanisms relevant to cell mobilization.

A potential mechanism explaining the positive effect observed in this study on gut health and performance might be related to the production of SCFA [76]. It has been extensively described in the literature that XOS influence the microbiome and favours the production of SCFA [16, 50, 77, 78]. Studies in humans indicate that SCFA are involved in proliferation and differentiation of epithelial cells and have been described to influence epithelial cell migration relevant for cell restitution after injury [79–81]. According to Park [82], Gram-positive bacteria are involved in the turnover of cells in the intestinal epithelium, an activity that is believed to be facilitated by SCFA. However, it is unclear that XOS-associated SCFA production in the chicken occurs in functional amounts in the jejunum. Thus, changes in XOS-associated microbial SCFA production and their possible effect relevant to intestinal epithelial cell turnover warrants further investigation.

Overall, XOS/AXRF supplementation leads to a phenotype which is consistent with an improved gut integrity and gut health requiring lower cell differentiation and migration. This improvement in intestinal health results in reduced energy requirements for cell differentiation and turnover, ultimately leading to more efficient growth.

## Conclusion

In conclusion, dietary supplementation of XOS/AXRF improved feed efficiency in broiler chickens fed a corn/soybean meal-based diet. The improved FCR was associated to a decrease in epithelial cell turnover and migration in the jejunum, which explains a reduction in the maintenance energy requirements of the GIT in approximately 19%. The results revealed that the dietary supplementation decreased the abundance of around 300 proteins (DAP), which in turn downregulated some key pathways such as the “actin filament polymerization” and the “oxidation-reduction process” pathways indicating a decrease in epithelial cell migration and a lower energy metabolism in the jejunum. The present study provides a comprehensive reference point whereby dietary XOS and AXRF supplementation can improve performance of chickens and a fundamental insight into the molecular mechanisms involved.

### Supplementary Information

**Additional file 1: Fig. S1.** Complete list of enriched pathways and differentially abundant proteins in supplemented chickens compared to control.

## Data Availability

The data presented in this study are available on request from the corresponding author.

## Abbreviations

ACN: Acetonitrile
ADFI: Average daily feed intake
ADG: Average daily gain
AX: Arabinoxylan
AXRF: Arabinoxylan-rich fraction
DAP: Differentially abundant proteins
DDA: Data dependent acquisition
DTT: Dithiothreitol
FA: Formic Acid
GIT: Gastrointestinal tract
GO: Gene Ontology
KEGG: Kyoto Encyclopedia of Genes and Genomes
SCFA: Short-chain fatty acid
SWATH-MS: Sequential Window Acquisition of all Theoretical Mass Spectra
XOS: Xylo-oligosaccharides
