# Supplementary material for: Dietary xylo-oligosaccharides and arabinoxylans improved growth efficiency by reducing gut epithelial cell turnover in broiler chickens
Source: J Anim Sci Biotechnol. 2024 Mar 4;15:35. doi: 10.1186/s40104-024-00991-z (PMC10910751; doi:10.1186/s40104-024-00991-z)
Supplement: Supplementary file 1 — Additional file 1: Fig. S1. Complete list of enriched pathways and differentially abundant proteins in supplemented chickens compared to control. [file 40104_2024_991_MOESM1_ESM.docx]

**Additional file 1: Fig. S1.** Complete list of enriched pathways and differentially abundant proteins in supplemented chickens compared to control.

| Database | Pathway | *P* value | Differentially abundant proteins |
| --- | --- | --- | --- |
| Gene Ontology  (Biological process) | Actin filament polymerization | 0.0000 | RAC1, ARPC4, COTL1, VIL1, GSN, CTTN |
|  | Oxidation-reduction process | 0.0003 | GLRX, ALDH1A1, LDHA, CYP2C23a, BCMO1, DCXR, AKR1A1, PRDX1, TXN, ADH1C |
|  | Prostaglandin metabolic process | 0.0014 | ACOX1, HPGDS, HPGD |
|  | Iron ion homeostasis | 0.0015 | BDH2, MELTF, SOD2, TF |
|  | Barbed-end actin filament capping | 0.0015 | HSPB1, VIL1, GSN, CAPZA2 |
|  | Protein folding | 0.0015 | PDIA6, TCP1, HSPA9, GRPEL1, CCT4, TXN, CCT8, ERP29 |
|  | Respiratory electron transport chain | 0.0020 | NDUFA5, SLC25A4, SOD2, ALDH5A1 |
|  | Gluconeogenesis | 0.0020 | TPI1, GOT2, FBP1, GPD2, PGK1 |
|  | Fatty acid beta-oxidation using acyl-CoA dehydrogenase | 0.0032 | ACOX1, ACAD9, ACADL, ETFA |
|  | ATP synthesis coupled proton transport | 0.0039 | ATP5H, ATP5F1, ATP8, ATP5B |
|  | Cell redox homeostasis | 0.0042 | GLRX, PDIA6, NNT, AIFM1, PRDX1, TXN |
|  | Actin filament severing | 0.0046 | VIL1, GSN, DSTN |
|  | Lipid homeostasis | 0.0056 | ACOX1, ACAD9, ACADL, ETFA |
|  | Positive regulation of protein localization to Cajal body | 0.0068 | TCP1, CCT4, CCT8 |
|  | Glutathione metabolic process | 0.0077 | IDH1, GSTA3, SOD2, ALDH5A1 |
|  | Vesicle-mediated transport | 0.0080 | RAB10, ARF1, SAR1B, CLTC, COPA, STXBP5L |
|  | Metabolic process | 0.0080 | ACSL5, GSTA3, ISOC1, GSTM2, LOC100859645, PNAT3 |
|  | Actin cytoskeleton reorganization | 0.0092 | RAC1, MYH9, CTTN, EZR, ANXA1 |
|  | Positive regulation of establishment of protein localization to telomere | 0.0093 | TCP1, CCT4, CCT8 |
|  | Toxin transport | 0.0117 | HSPA5, TCP1, CCT4, CCT8 |
|  | Fatty acid beta-oxidation | 0.0117 | ACOX1, BDH2, EHHADH, ECI1 |
|  | Actin filament-based movement | 0.0191 | MYH9, MYO6, MYO1A |
|  | Positive regulation of establishment of protein localization to telomere | 0.0093 | TCP1, CCT4, CCT8 |
|  | Intracellular protein transport | 0.0277 | SEC24C, CTTN, MYO6, SAR1B, AP2M1, CLTC, COPA, ERP29 |
|  | Glyceraldehyde-3-phosphate biosynthetic process | 0.0433 | TPI1, TKT |
|  | Isocitrate metabolic process | 0.0433 | IDH1, IDH2 |
|  | Regulation of cell-cell adhesion mediated by integrin | 0.0433 | ADA, DPP4 |
|  | Positive regulation of protein processing in phagocytic vesicle | 0.0433 | MYH9, GSN |
|  | Cytoplasmic translation | 0.0468 | RPL31, EIF4H, RPL6 |
|  | Epithelial cell differentiation | 0.0499 | ANXA4, BDH2, VIL1, HNRNPH3 |
| KEGG | Metabolic pathways | 0.0000 | CMPK1, FDPS,NANS, PKM, DCXR, GOT2, MDH2, LTA4H,ATP5B, DAK,CPOX, ACOX1, ATP5F1,OGDH, CYP3A80, LOC100857622, HSD11B1b, PGLS, LDHA, COX4I1, 421152, FBP1, GK, BDH2, NDUFA9, ATP5H, UQCRC2, ADA, ALDH5A1, 771920,TKT, ALDH1A1, CKMT1A, TPI1, HPGDS, 395346, HSD17B4, IDH1, ACO1, AKR1A1, IDH3B, IMPA1,ACO2, ATP5J2, ACSL5, HSD17B10, ATP8,PPCS, ATP5D, ASAH1, UQCRC1,IDH2, HEXA, NNT, CKB, COX6A1, CYP3A7, UGT2A1, PGK1, LOC425137, ACADL, SPR, AGMAT, PNAT3, 417907,PDHB, NDUFA5, SI, EHHADH, PGD, MTHFD1, ALPP, MAOA |
|  | Carbon metabolism | 0.0000 | TPI1, PKM, GOT2, IDH1, MDH2, FBP1, ACO1, PGK1, IDH3B, ACO2, DAK, PDHB, OGDH, EHHADH, PGD, TKT, IDH2, PGLS |
|  | Biosynthesis of antibiotics | 0.0000 | PGLS, FDPS, LDHA, TPI1, PKM, GOT2, IDH1, MDH2, FBP1, ACO1, AKR1A1, PGK1, IDH3B, ACO2, PDHB, HSD17B10, OGDH, EHHADH, PGD, TKT, IDH2 |
|  | Metabolism of xenobiotics by cytochrome P450 | 0.0000 | EPHX1L, MGST3, UGT2A1, GSTA3, GSTM2, DHDH, LOC100859645, HSD11B1b, ADH1C |
|  | Citrate cycle (TCA cycle) | 0.0000 | PDHB, OGDH, IDH1, ACO1, MDH2, IDH3B, IDH2, ACO2 |
|  | 2-Oxocarboxylic acid metabolism | 0.0000 | GOT2, IDH1, ACO1, IDH3B, IDH2, ACO2 |
|  | Biosynthesis of amino acids | 0.0001 | TPI1, GOT2, PKM, IDH1, ACO1, PGK1, IDH3B, TKT, IDH2, ACO2 |
|  | Oxidative phosphorylation | 0.0001 | COX4I1, COX6A1, NDUFA12, ATP5B, ATP5J2, PPA2, NDUFA9, NDUFA5, ATP5H, ATP5F1, UQCRC2, ATP8, UQCRC1, ATP5D |
|  | Glutathione metabolism | 0.0003 | GPX3, MGST3, IDH1, GSTA3, PGD, GSTM2, LOC100859645, IDH2 |
|  | Drug metabolism - cytochrome P450 | 0.0003 | MGST3, UGT2A1, GSTA3, GSTM2, LOC100859645, ADH1C, MAOA |
|  | Retinol metabolism | 0.0005 | ALDH1A1, BCMO1, CYP3A7, UGT2A1, SDR16C5, CYP3A80, ADH1C |
|  | Peroxisome | 0.0012 | ACOX1, ACSL5, HSD17B4, EHHADH, IDH1, SOD2, HACL1, PRDX1, IDH2, SLC27A2 |
|  | Glycolysis / Gluconeogenesis | 0.0016 | PDHB, LDHA, TPI1, PKM, FBP1, AKR1A1, PGK1, ADH1C |
|  | Pentose and glucuronate interconversions | 0.0023 | UGT2A1, DCXR, AKR1A1, LOC425137, DHDH |
|  | PPAR signaling pathway | 0.0038 | ACOX1, FABP3, DBI, GK, ACSL5, EHHADH, ACADL, SLC27A2 |
|  | Fatty acid degradation | 0.0049 | ACOX1, ACSL5, EHHADH, ECI1, ACADL, ADH1C |
|  | Steroid hormone biosynthesis | 0.0281 | CYP3A7, UGT2A1, SULT1E1, CYP3A80, HSD11B1b |
|  | Pentose phosphate pathway | 0.0382 | FBP1, PGD, TKT, PGLS |
| Reactome | Citric acid cycle (TCA cycle) | 0.0008 | NNT, OGDH, MDH2, IDH2,ACO2 |
|  | Gluconeogenesis | 0.0029 | TPI1, GOT2, FBP1, MDH2, PGK1 |
|  | Glutathione conjugation | 0.0035 | HPGDS, MGST3, GSTA3, AKR1A1 |
|  | RHO GTPases activate KTN1 | 0.0099 | RAC1, CDC42, KTN1 |
|  | Nonsense Mediated Decay (NMD) independent of the Exon Junction Complex (EJC) | 0.0149 | RPS16, RPL31, RPS10, RPSAP58, RPL6, RPS7, RPL27A |
|  | Glycolysis | 0.0194 | TPI1, PKM, PGK1, PFKP |
|  | Association of TriC/CCT with target proteins during biosynthesis | 0.0200 | TCP1, CCT4, CCT8 |
|  | Factors involved in megakaryocyte development and platelet production | 0.0208 | RAC1, HBBA, PRKAR2A, CDC42, LOC417953, CAPZA2 |
|  | Gluconeogenesis | 0.0259 | TPI1, GOT2, FBP1, PGK1 |
|  | Detoxification of Reactive Oxygen Species | 0.0295 | GPX3, SOD2, PRDX1, TXN |
|  | Nonsense Mediated Decay (NMD) enhanced by the Exon Junction Complex (EJC) | 0.0309 | RPS16, RPL31, RPS10, RPSAP58, RPL6, RPL27A, RPS7 |
|  | Gap junction degradation | 0.0328 | MYO6, AP2M1, CLTC |
|  | Ribosomal scanning and start codon recognition | 0.0341 | RPS16, RPS10, RPSAP58, EIF4H, RPS7 |
|  | Respiratory electron transport | 0.0371 | NDUFA9, NDUFA5, UQCRC2, NDUFA12, UQCRC1 |
|  | RHO GTPases activate PAKs | 0.0402 | RAC1, CDC42, MYLK |
|  | RHO GTPases Activate WASPs and WAVEs | 0.0464 | RAC1, CDC42, ARPC4, ACTR2 |
